# Depression in youths with early life adversity: a systematic review and meta-analysis

**DOI:** 10.3389/fpsyt.2024.1378807

**Published:** 2024-09-12

**Authors:** Zengyan Yu, Yunhua Cao, Tinghuizi Shang, Ping Li

**Affiliations:** Department of Psychiatry, Qiqihar Medical University, Qiqihar, China

**Keywords:** depression, adversity, threat, deprivation, youth

## Abstract

**Background:**

Globally, early-life adversity (ELA) is linked to an increased risk of developing depression in adulthood; however, only a few studies have examined the specific effects of various types of ELA on depression in children and adolescents. This meta-analysis explores the association between the subtypes of ELA and the risk for youth-onset depression.

**Methods:**

We searched three electronic databases for reporting types of ELA, namely, emotional abuse, physical abuse, sexual abuse, emotional neglect, physical neglect, family conflict/violence, divorce, low socioeconomic status, and left-behind experience, associated with depression before the age of 18 years. Our meta-analysis utilized the odds ratio (OR) and relied on a random effects model. Large heterogeneous effects were detected. Some factors moderated the association between ELA and depression in youths. The homogeneity of variance test and meta-regression analysis were used to detect these relationships.

**Results:**

A total of 87 studies with 213,006 participants were ultimately identified via several strategies in this meta-analysis. Individuals who experienced ELA were more likely to develop depression before the age of 18 years old than those without a history of ELA (OR=2.14; 95% CI [1.93, 2.37]). The results of the subgroup analysis revealed a strong association between ELA and depression in youth, both in terms of specific types and dimensions. Specifically, emotional abuse (OR = 4.25, 95% CI [3.04, 5.94]) was more strongly related to depression in children and adolescents than other forms of ELA were. For both dimensions, threat (OR = 2.60, 95% CI [2.23, 3.02]) was more closely related to depression than deprivation was (OR = 1.76, 95% CI [1.55, 1.99]).

**Conclusion:**

This meta-analysis revealed that the adverse effects of a broader consideration of ELA on the risk of youth-onset depression vary according to the subtypes of ELA.

**Systematic review registation:**

https://www.crd.york.ac.uk/prospero/display_record.php?ID=CRD42023405803, identifier 42023405803.

## 1 Introduction

According to the World Health Organization in 2019, depression is the fourth leading cause of illness and disability in children and adolescents, seriously affecting their physical health and academic life and further placing a heavy economic burden on families and society (1). The prevalence of depression in children and adolescents has reached 15%-20% (2). In particular, in recent years, the COVID-19 pandemic has had a more serious impact on children and adolescents with mood disorders (3, 4). According to the 2022 National Depression Blue Book, the global burden of mental disorders has increased since the pandemic, with a surge of 53 million patients with depression (an increase of up to 27.6%), with 50% of patients with depression being school-age students (5). For these reasons, identifying the risk factors for depression in children and adolescents is a key issue for prevention and intervention. Some relatively common risk factors for depression in young individuals include genetic factors, such as temperament (6), and environmental factors, such as early-life adversity (7). In recent years, interest in exploring the impact of early-life adversity on depression in children and adolescents in terms of cumulative risk to the family has been growing (8).

Early life adversity (ELA) refers to the adverse environment experienced by individuals in their early years (infancy, childhood, and adolescence) and may be a significant risk factor for depression in children and adolescents (9, 10). More than half of youth have experienced at least one form of ELA (e.g., abuse, neglect, poverty, or loss of a parent), and youths who experience ELA develop an increased risk for mood disorders by age 18 (11). Recent meta-analyses suggest that ELA is linked to a twofold increase in the risk for major depressive disorder in adolescents (7). In addition, some studies have shown that ELA can be further categorized into two distinct subtypes of threat and deprivation (12), with threat referring to life-threatening, injurious, sexually assault, or other harm to an individual’s physical integrity, and deprivation primarily refers to a lack of expected environmental input in the cognitive (e.g., language) and social domains and a lack of complexity of environmental stimuli appropriate to the species and age (12). Based on threat and deprivation typology is a novel conceptual framework for examining the impact of early family adversity on an individual’s neurodevelopment, which in turn leads to different forms and degrees of physical and psychological problems (12, 13). Subtyping is thus more useful for synthesizing and quantitatively analyzing the relationship between ELA and adolescent depression.

While these studies provide valuable information, certain limitations are noteworthy. First, previous meta-analytic studies have focused primarily on adult populations (14, 15), with less focus on exploring the effects of ELA on MDD in adolescent populations. This is pertinent because the etiology, clinical presentation, and course of depression differ between adolescents and adults (16), and it is reasonable to assume that the nature of the relationship between ELA and depression may also vary depending on the stage of development (7). Second, previous meta-analytic studies on the relationship between ELA and depression have shown a more homogenous form of ELA, focusing mainly on childhood traumatic experiences (17). Based on the cumulative risk model and the family stress model, the measures of family risk, in addition to family climate risk (e.g., child abuse and neglect, domestic violence), family structural risk (e.g., divorce), and family resource insufficiency risk (e.g., poverty), are underexplored in terms of their relationship with depression (18). Third, the findings of the few studies on ELA and adolescent depression are not entirely consistent with each other; this is due not only to the choice of the specific form of ELA or its measurement (7) but also to the small number of studies that have opted to enter the meta-analysis, limiting the scope of the analysis of the moderating effects that influence the relationship.

In summary, the current study explored the relationships between the nine specific forms of ELA (including sexual abuse, physical abuse, psychological abuse, physical and emotional neglect, divorce, being left behind, low socioeconomic status, and domestic violence) and its subtypes (threat and deprivation) and risk for youth-onset depression via meta-analysis, as well as the moderating variables affecting the relationship between ELA and depression. The meta-analytic technique not only integrates the results of multiple studies and effectively reduces the measurement and sampling errors that exist in the results of a single study but also helps identify the extent to which different ELA experiences impact depression based on a quantitative review of many research results and a comprehensive analysis technique to provide a certain guiding value for the intervention of clinical mood disorders. Specifically, this study used meta-analytic techniques to answer the following two questions: first, to what extent do the nine ELA experiences and two subtypes influence depression? Second, what demographic and/or methodological factors moderate the association between ELA and depression?

## 2 Methods

This meta-analysis followed the Preferred Reporting Items for Systematic Reviews and Meta-Analyses (PRISMA) guidelines (19), and the protocol was registered in the PROSPERO system (registration number CRD42023405803).

### 2.1 Inclusion and exclusion criteria

The studies included the following criteria in this meta-analysis: (1) operational definition of ELA in the research included nine specific forms (emotional abuse, physical abuse, sexual abuse, emotional neglect, physical neglect, family conflict/violence, divorce, low socioeconomic status, and left-behind experience); (2) depression prior to a mean age of 18 years old and evaluation of relevant depression measures; (3) empirical studies must have reported numerical results, and the effect sizes of the relationships between subtypes of ELA and depression were directly obtained; and (4) selection of only one article when multiple articles were published using the same set of data. The exclusion criteria were as follows: (1) studies on adult samples; (2) the nine specific ELA types were not mentioned in the study or mixed together (e.g., childhood maltreatment) and could not be obtained in relation to depression; (3) lack of sufficient data needed to calculate effect size; and (4) conference abstracts with no empirical data (e.g., reviews, editorials).

### 2.2 Search strategy

**Figure 1:**
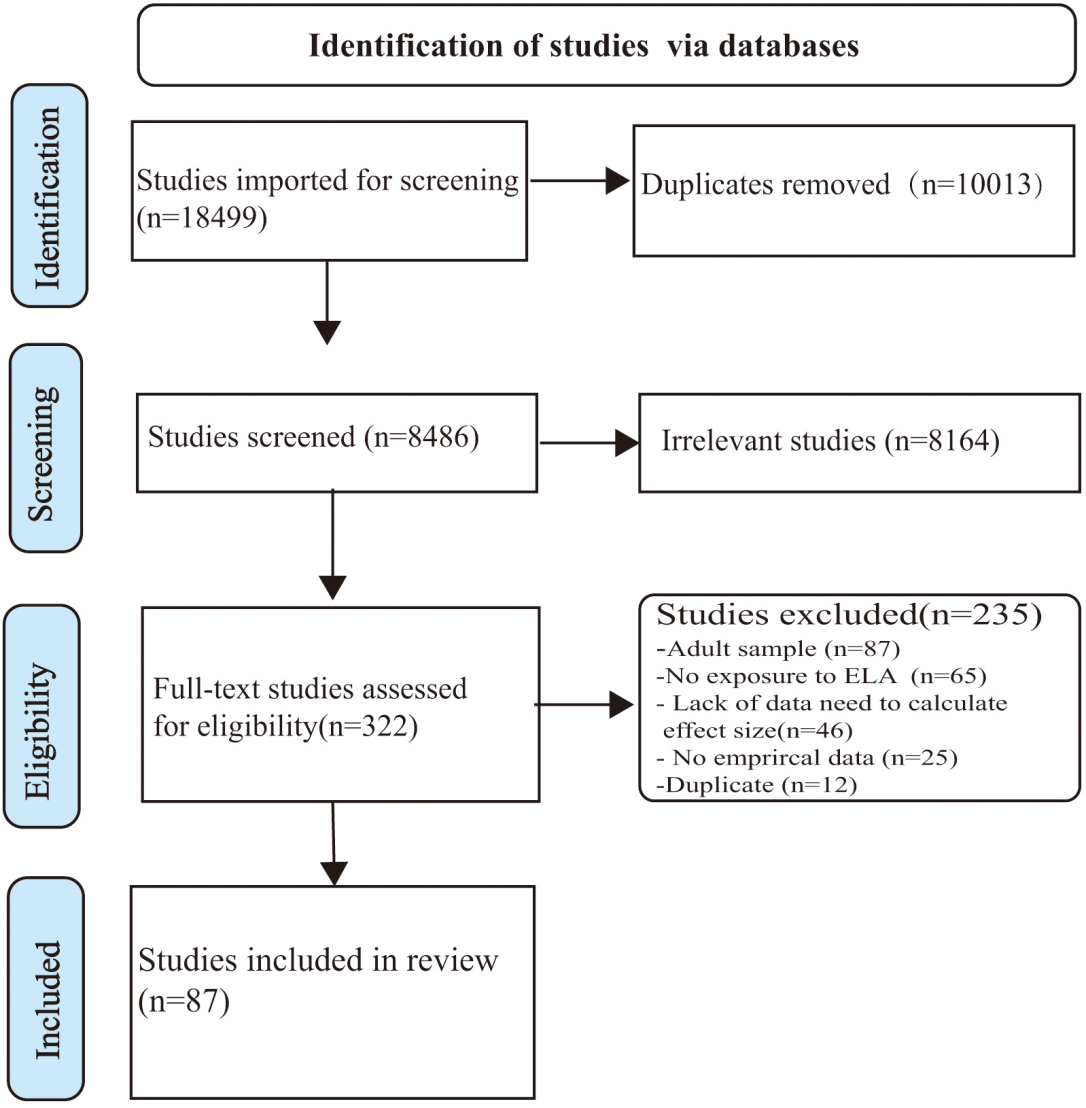
PRISMA Flowchart of Study Selection.

First, we conducted computer-based searches in the Web of Science and PubMed for articles published in English and the CNKI Database for articles published in Chinese from inception to December 2022. These articles were searched using the following keywords (or stems): (“Affective Disorder” OR “Mood Disorder” OR “Depressive symptoms” OR Depress OR MDD) AND (Child OR Childhood OR Children OR Adolescent OR Adolescence) AND (“Early life stress” OR “Early life adversity” OR “Adverse childhood experiences” OR Maltreatment OR “Physical abuse” OR “Sexual abuse” OR “Emotional abuse” OR “Psychological abuse” OR Trauma OR Neglect OR “Domestic violence” OR Divorce OR “Socioeconomic status” OR Left-behind). Second, we consulted the bibliography using both forward and backward searches to find additional studies, especially relevant meta-analyses and systematic reviews. The early life adversity subtypes were we have checked they are diplayed correctly clearly defined in this meta-analysis (see **Supplementary 1**). The results are outlined in the PRISMA flow chart (**Figure 1**).

### 2.3 Data extraction

Duplicate documents were removed using Endnote X9 and then independently screened by two authors. The effect sizes, moderator codes, and study quality assessments were extracted with essentially the same results. Disagreements were resolved in consensus meetings, and the lead authors made a final determination. Rater agreement at the screening stage was 96%.

### 2.4 Moderator variables of encoding

The studies included in the meta-analysis were coded as follows when available (see **Table 1**). Study information (first author + publication year), sample size, female rates, average age, sample source (school, community, services, clinic/hospital), country (countries were coded as developing or developed for data analysis), assessment tools of depression, research design (cross-sectional, case-control, and cohort study), and subtypes of ELA (dimensions: threat and deprivation; the former in this study included emotional abuse, physical abuse, sexual abuse, and family violence, and the latter mainly included low socioeconomic status, divorce, and being left with two parents away).

**Table 1:** Characteristics of Studies Included in the Meta-Analysis.

Study	Sample Size	Age (*M*)	% Female Participants	Sample Source	Country	Depression Assessment	ELA Type	Research design	Quality Assessment
Adams et al. (20)	3,424	14.50	49%	Community	USA	NSA-R	DV	A	11
Ahmadkhaniha et al. (21)	87	10.98	36%	Community	Iran	K-SADS	SA	A	11
Avanci et al. (22)	464	8	48	School	Brazil	CBCL	Divorce, DV, EA, Low SES, PA	A	8
Bielas et al. (23)	130	16.84	0%	Juvenile detention centre	Switzerland	MINI-KID	Low SES	A	11
Brown et al. (24)	639	17.99	48%	Random sample	New York state	NIMH-DISC	PA, SA, EN	B	8
Calvete (25)	1052	13.43	47%	School	Spain	CES-D	EA	B	8
Cao et al. (26)	724	NS	49%	School	China	CES-D	Low SES	A	7
Carey et al. (27)	94	14.25	63%	Trauma clinic	South Africa	K-SADS	SA	A	9
Castro et al. (28)	124	10.58	63%	Community	USA	CDI	Low SES	A	8
Chen and Chan (29)	793	13.96	48	School	China	CES-DC	Left- behind	A	6
Cohen et al. (30)	105	14.70	70%	Psychiatric inpatient program	USA	DICA-R and clinical evaluation conference	PA, SA	A	11
Courtney et al. (31)	195	16.3	79	Clinic	USA	BDI	EA	A	6
Danielson et al. (32)	548	15.03	64	Community	USA	NS	PA	A	7
Daryanani et al. (33)	287	14	50	Community	USA	K-SADS-E	Low SES	B	5
Daviss et al. (34)	104	13.78	37%	Mental health clinic and community	USA	K-SADS-PL	SA, PA, DV	A	7
Dhamayanti et al. (35)	786	13	44	School	Indonesia	CDI	EA	A	7
Dunlop and Burns (36)	80	15.00	48%	Community	Australia	NSQ	Divorce	B	6
Dunn et al. (37)	3686	15.95	NS	School	USA	CES-D	PA, SA	B	7
Elmore and Crouch Amanda (38)	39929	12.50	49%	Community	USA	NS	Divorce, DV	A	10
Fang (39)	2,614	15.76	54%	School	China	SCL-90	Divorce	A	6
Farrell et al. (40)	878	10.58	48%	School	Australia	CDI	Low SES	A	5
Fergusson et al. (41)	935	15	50	Community	New Zealand	DSM-III-R	Divorce	B	7
Flisher et al. (42)	665	13.10	52%	Community	USA	NIMH-DISC	PA	A	9
Gallo et al. (43)	1954	18	99	Clinic	Brazil	MINI V5.0	DV, EA	B	9
Gilman et al. (44)	1,089	13.99	47%	Community	RI	DIS	Low SES, Divorce	B	7
Goodman (45)	15483	15.9	48.9	Community	USA	CES-D	Low SES	B	2
Goodman et al. (46)	15112	16.1	48.8	Community	China	CES-D	Low SES	A	5
Goodman et al. (47)	13235	15.9	48.8	School	USA	CES-D	Low SES	B	7
Greger et al. (48)	335	16.85	59%	Residential child welfare institutions	Norway	CAPA	SA, PA, DV	A	11
Guo et al. (49)	3759	12.64	48	School	China	CDI-S	Left- behind	A	5
Guo et al. (50)	3254	12.56	44	School	China	CDI-S	Left- behind	C	6
Hanson et al. (51)	3,906	14.49	49%	Community	USA	NWS Depression Modules	SA, PA, DV	A	11
He et al. (52)	875	11.05	37%	School	China	CDI	Left-behind	A	8
He (53)	655	14.56	48%	School	China	CES-D	Low SES	A	5
Horesh et al. (54)	40	17.12	55%	Psychiatric clinic	Sweden	K-SADS-H	SA	C	7
Jaffee et al. (55)	998	15.00	48%	Community	New Zealand	NIMH-DISC	SA	B	8
Ji et al. (56)	2,805	14.25	55%	School	China	CES-D	Left-behind	A	8
Kaplan et al. (57)	198	15.00	50%	Department of Social Services	NY	K-SADS-E	PA	C	9
Kaufman (58)	56	9.58	52%	Department of Children and Youth Services	USA	K-SADS-P	PA,EA,SA	A	9
Kerr and Beer (59)	122	12.10	45%	School	USA	BDI	Divorce	A	8
Kilic et al. (60)	121	15.18	49%	Psychiatric outpatient unit	Turkey	K-SADS-PL	PA,EA,SA	C	5
Kolko et al. (61)	103	9.90	27%	Child psychiatric unit	USA	NS	PA,SA	A	11
Kuyken et al. (62)	50	15.92	82%	Clinical, school, community sources	U.K.	SCID	SA	C	8
Lewis et al. (63)	427	14.6	54	Community	USA	CDRS-R	PA	B	8
Lin et al. (64)	881	9.61	49.6	School	Taiwan,China	CDI	Low SES	A	10
Ling et al. (65)	1,219	16.67	49%	School	China	CES-D	EA, PA,PN,EN	A	7
Liu and Zhang (66)	715	14.09	52%	School	China	CES-D	EA, PA,SA,PN,EN, Left-behind	A	6
Mac Giollabhui et al. (67)	173	12.5	56%	Community sample	USA	K-SADS-E	EA	B	7
Mansbach et al. (68)	906	13.5	50	Community	Israel	DAWBA	SA	A	7
McLeer et al. (69)	49	9.66	51%	Psychiatric outpatient clinic	Pennsylvania	K-SADS-E	SA	C	9
Monteiro et al. (70)	319	13.94	68	Community	Portugal	CDI	EA,EN	A	5
Morais et al. (71)	498	15.93	0%	Residential sex offender treatment facility	USA	K-SADS-PL	SA	A	11
Moretti and Craig (72)	179	15.34	46%	Centers servicing youth	*Canada*	OCHS	Child abuse	B	7
Münzer et al. (73)	178	11.51	45	Services	Germany	Kiddie-SADS	SA	A	9
Myers (74)	188	17.00	60%	School	USA	CES-D	Low SES	A	8
Olsson (75)	150	16.50^c^	77%	High-schools	Sweden	DICA-R	PA, DV	C	10
Pantle and Oegema (76)	111	15.60	100%	Hospital	MI	NS	SA	C	10
Pelcovitz et al. (77)	185	15.16	52%	Department of Social Services	NY	K-SADS-E	DV	C	10
Pham et al. (78)	546	14.9	59	Community	Vietnam	CES-D	EA,EN	A	6
Phillips et al. (79)	630	14.90	49%	Community	Australia	K-SADS-E	Low SES	B	7
Poulsen et al. (80)	2581	15	50.5	Community	Denmark	CES-DC	Low SES	B	6
Qu (81)	2,105	12.31	45%	School	China	CES-D	EA, PA,PN,ENDV, Divorce Low SES	B	7
Rizzo et al. (82)	155	15.00	76%	Psychiatric inpatient unit	USA	K-SADS-PL	EA	A	9
Rønning et al. (83)	2348	8	0	Community	Finnish	CDI、BDI	Divorce	B	8
Sadowski et al. (84)	46	10.30	100%	Clinic/hospital	U.K.	K-SADS	SA	A	9
Sandler et al. (85)	168	11.47	48.8	Community	China	CAS	Divorce	B	7
Shah et al. (86)	518	14.3	61	School	United Arab Emirates	BDI	EA,PA	A	6
Shanahan et al. (87)	1,004	12.50	44%	Community	USA	CAPA	Low SES, Child abuse	B	7
Shen et al. (88)	2283	14.22	55	School	China	CDI	Left- behind	A	6
Shi et al. (89)	2968	18.27	39	School	China	SCL-90	Left- behind	A	7
Størksen et al. (90)	2171	14.5	53.9	School	Norway	SCL-5	Divorce	B	7
Sun (91)	397	16.95	60%	School	China	CDI	Low SES	A	**5**
Sun et al. (92)	1,230	10.45	47%	School	China	CDI	Left-behind	A	**5**
Tang et al. (93)	1,000	9.5	48%	School	China	CES-D	Left-behind	A	6
Tummala-Narra and Sathasivam-Rueckert (94)	707	14.85	51%	School	USA	CES-D	Low SES	A	8
Wahab et al. (95)	51	15.06	100%	Clinic/hospital	Malaysia	K-SADS-PL	SA	A	9
Wilson et al. (96)	1,698	16.99	54%	Community	USA	DICA-R	PA, SA	B	8
Wu et al. (97)	879	NS	NS	School	China	CES-D	Left-behind	A	7
Xiao et al. (98)	1134	13.47	74	School	China	K-SADS-PL	EA,PA,PN	C	7
Yen et al. (99)	1684	14.4	51	School	China	ZDS	PA	A	8
Yin et al. (100)	437	14.95	49%	School	China	CES-D	Low SES	A	5
Yu et al. (101)	687	16.44	64	School	China	CES-D	EA	A	5
Zhang et al. (102)	47872	14.11	53.6	School	China	CES-D	Divorce	A	5
Zhang (103)	638	11.18	46%	School	China	CDI	Low SES	B	6
Zhang et al. (104)	6,228	13.96	52%	School	China	SDS	EA, PA,SA,PN,EN	A	7
Zhao (105)	1,462	15.18	52%	School	China	SDS	Low SES	A	6
Zou et al. (106)	652	14.55	48%	School	China	CES-D	Low SES	A	7

ELA, Adverse childhood experiences; DV, Domestic violence; SA, Sexual abuse; PA, Physical abuse; EA, Emotional abuse; EN, Emotional neglect; PN, Physical neglect; Low SES, Low so-economic status; CES-D, Center for Epidemiological Studies Depression Scale; SDS, Self-rating depression scale; CDI, Children’s Depression Inventory; BDI, Back Depression Inventory; NSA, National Survey of Adolescents (R, Replication); K-SADS, Kiddie Schedule for Affective Disorders and Schizophrenia; (PL, Present and Lifetime Version; E, Epidemiological Version); MINI-Kid, Mini-International Neuropsychiatric Interview for Children and Adolescents; NIMH-DISC, National Institute of Mental Health Diagnostic Interview Schedule for Children; DICA, Diagnostic interview for children and adolescents; (R, Revised); NSQ, Neuroticism Scale Questionnaire; NS, Not Specified; DIS, Diagnostic Interview Schedule; CAPA, Child and Adolescent Psychiatric Assessment; SCID, Structured Clinical Interview for DSM; DSRSC, Depression Self-rating Scale for Children; OCHS, Ontario Child Health Study-Youth Self-report; NWS, National Women’s Study; A, Cross-sectional study; B, Cohort study; C, Case-control.

In terms of the number of individual studies by country, China (n=29) and the United States (n=27) accounted for the majority of studies, and most of the remaining countries only had one or two studies. Depression measurement tools were dominated by the CES-D (n=22), CDI (n=13), and K-SADS (n=16), while the use of the BDI, CBCL, SCL-90, and other instruments was less relevant. The research methods used were mostly cross-sectional studies (n=54), whereas cohort studies (n=23) and case−control studies (n=10) were relatively rare.

### 2.5 Quality evaluation

The studies included in this meta-analysis included cross-sectional studies, cohort studies, and case-control studies. We coded study quality using the Agency for Healthcare Research and Quality (AHRQ) to assess cross-sectional studies, and the AHRQ score was 11 points. Each entry is given a score of 1 point, with 8 points or more indicating high quality, 4-7 points indicating medium quality, and 0-3 points indicating low quality (107). We used the Newcastle Ottawa Scale (NOS) to assess cohort studies and case−control studies, with NOS scores of 9 points (108). A score of 7 or higher indicates high quality, 4-6 indicates medium quality, and 0 to 3 indicates low quality (see **Table 1**).

### 2.6 Statistical analysis

#### 2.6.1 Calculation of effect sizes

The correlation coefficient (r), standardized mean difference (d), or odds ratio (OR) for the relationship between adverse childhood experiences and depression in childhood or adolescence were reported for these 101 effect sizes. The extracted data were converted to OR effect sizes, which were used to integrate the relationship between ELA and depression in children and adolescents. We used CMA 2.0 to conduct statistical analyses, which allowed the direct input of multiple effect sizes, which can be converted into ORs (109). First, the effect size r is converted to d, and then d is converted to OR. The conversion formula is as follows:


$$\text{d}=\frac{2\text{r}}{\sqrt{1-{\text{r}}^{2}}};\text{ logOR}=\frac{\text{π}\times \text{d}}{\sqrt{3}};\text{ OR }=\text{ Exp}\left(\text{logOR}\right).$$

*Heterogeneity test* The standard Cochran Q test (calculation of *I* ^2^-value) was used to test the heterogeneity of the effect size. When *I* ^2^ ≧ 50, indicating the presence of heterogeneity, a random effects model was chosen (110).

#### 2.6.2 Publication bias test

Publication bias may severely affect the results of the meta-analysis. Generally, the publication bias of a meta-analysis is comprehensively evaluated using funnel plots, the classic fail-safe N test (no publication bias when the N value is greater than 5k+10), and Egger’s test (no publication bias when the intercept in the regression equation is zero). Among the three methods, Egger’s test was relatively more objective and accurate in assessing publication bias.

#### 2.6.3 Meta-analysis procedure

First, the relationships between the different forms of ELA and depression in children and adolescents were explored by meta-analytic techniques, and then, demographic and methodological factors were tested to determine whether they moderated the association between ELA and depression. Specifically, the homogeneity of variance test in the *Q* test was used for categorical moderated variables, and the moments of random effects model regression analysis were used for continuous variables.

## 3 Results

### 3.1 ELA (all forms) and depression

In this meta-analysis, a total of 87 studies with 136 effect sizes and 213,006 unique individuals were included to examine the relationship between ELA and depression in children and adolescents (see **Table 1**), with a broad range of ORs (see **Supplementary Figures S1–S9**). Random effects meta-analysis showed that those who experienced ELA are more likely to suffer from depression in childhood or adolescence than those with no history of ELA (*OR* = 2.14, 95% *CI* = 1.93, 2.37), an effect that differed significantly from zero (*Z* = 14.65, *p* < 0.001). There was significant heterogeneity across studies (*Q*_100_ = 2740.73, *p* < 0.001, *I* ^2^ = 95.04%), and we further conducted moderation effect analysis (see **Table 2**).

**Table 2:** Results From Random-Effects Meta-analyses for Each Type of ELA Examined in Relation to depression.

ELA type	*k*	*OR* (95% *CI*)	*Z*	*Q*	*I* ^2^	Classic Fail-safe *N*	Egger’s Intercept	Moderators
SA	24	1.85(1.57,2.19)	7.26***	80.37***	70.14%	1259	0.22(-0.73,1.17)	publication year
PA	23	2.21(1.88,2.59)	9.60***	77.13***	71.48%	2232	-1.04(-2.21,0.14)	none
L-SES	25	1.57(1.40,1.75)	7.84***	106.59***	77.48%	1448	1.24(-0.07,2.54)	development level
EA	18	4.25(3.04, 5.94)	8.47***	372.24***	95.43%	6993	-2.41(-6.63,1.80)	none
DV	10	2.45(1.88,3.18)	6.66***	32.005***	71.88%	435	0.96(-1.47,3.40)	none
L-behind	12	1.50(1.31,1.72)	5.83***	38.79***	71.64%	334	2.25(-0.81,5.31)	Mean age (ELA time frame)
EN	7	3.09(1.75,5.47)	3.87***	242.600***	97.53%	1395	-4.54(-14.97,5.90)	none
Divorce	12	1.58(1.42,1.76)	8.41***	19.950*	44.86%	461	0.30(-1.06,1.66)	none
PN	5	2.25(2.00,2.52)	13.68***	8.36	52.13%	568	1.85(-2.32,6.01)	N/A
Threat	75	2.60(2.23,3.02)	12.27***	1180.48***	93.65%	35316	0.09(-1.22,1.39)	Publication year;
Deprivation	61	1.76(1.55,1.99)	8.82***	1194.86***	94.98%	9560	0.08(-1.88,2.04)	none
ELA(all forms)	136	2.14(1.93,2.37)	14.65***	2740.73***	95.04%	107578	0.40(-0.76,1.56)	publication year

SA, Sex Abuse; PA, Physical Abuse; L-SES, Low So-Economic Status; EA, Emotional Abuse; DV, Domestic Violence; L-behind, Left-behind; EN, Emotional Neglect; PN, Physical Neglect; N/A, not applicable given there was no significant heterogeneity; ELA, Early Life Adversity; *p <.05; ***p <.001.

### 3.2 Specific types of ELA and depression

**Table 2** presents the results of random-effects meta-analyses for each of the nine types of ELA examined in relation to the risk for depression. The forest plots are presented in **Supplementary Figures S1–S9**. Specifically, the risk factors for depression for each of the nine specific forms of early-life adversity, in order of magnitude, were emotional abuse (*OR* = 4.25, 95% *CI* = 3.04, 5.94), emotional neglect (*OR* = 3.09, 95% CI = 1.75, 5.47), domestic violence (*OR* = 2.45, *95% CI* = 1.88, 3.18), physical neglect (*OR* = 2.25, 95% *CI* = 2.00, 2.52), physical abuse (*OR* = 2.21, 95% *CI* = 1.88, 2.59), sexual abuse (*OR* = 1.85, 95% *CI* = 1.57, 2.19), low socioeconomic status (*OR* = 1.57, 95% *CI* = 1.40, 1.75), divorce (*OR* = 1.58, 95% *CI* = 1.42, 1.76), and being left behind (*OR* = 1.50, 95% *CI* = 1.31, 1.72) (see **Table 2**). Moreover, in terms of threat and deprivation, which are two types of ELA, threats had a more severe impact on depression in children and adolescents (*OR* = 2.60, 95% *CI* = 2.23, 3.02) than did the deprivation of adversity experiences (*OR* = 1.76, 95% *CI* = [1.55, 1.99]). Similarly, we also conducted a moderating effect analysis for each subtype of ELA in which there was significant heterogeneity.

### 3.3 Moderating effect test

We examined the role of each moderating variable separately in the relationship between all forms or subtypes of ELA and depression in children and adolescents (see **Table 2**). First, development level was coded as a dummy variable based on whether the level of socioeconomic development was indicative of a developing or developed country. Both sets of analyses revealed a statistically significant effect of development level on the association between low social status and depression in children and adolescents. Specifically, the studies that were based in developing countries (*k* = 11; *OR* =1.79; 95% *CI* = [1.60, 2.01]) had a larger estimated effect size than did the studies that included developed countries (*k* = 14; *OR* = 1.36; 95% *CI* = [1.18, 1.57]).

Second, there was a strong positive moderating effect of year of publication on ELA and youth-onset depression. The ELA variables involved were primarily all forms of ELA (*slope* = 0.02; 95% *CI* = [0.007, 0.028]; *Z* = 3.18; *p* < 0.01; *k* = 137), threat (*slope* = 0.025; 95% *CI* = [0.009, 0.041]; *Z* = 3.081; *p* < 0.01; *k* = 76), and SA (*slope* = 0.023; 95% *CI* = [0.006, 0.041]; *Z* = 2.635; *p* < 0.01; *k* = 25). Third, the ELA time frame had an important negative moderating effect on the relationship between being left behind and depression in children and adolescents. (*slope* = -0.054; 95% *CI* = [-0.105, -0.003]; *Z* = -2.072; *p* < 0.05; *k* = 12). The results revealed that the effects of parental absence or lack of resources on depression in children and adolescents weakened or diminished with age.

### 3.4 Heterogeneity test

The results of the heterogeneity test for the subtypes of ELA and depression in childhood or adolescence are shown in **Table 2**. The results of **Table 2** show that the *Q* test was significant, where *I* ^2^ > 50%, except divorce, indicating that there was substantial heterogeneity in the effect sizes of most of the studies in the meta-analysis and that the random effects model selected for the meta-analysis was accurate.

### 3.5 Publication bias test

First, a funnel plot was used to check for publication bias in the meta-analysis, as shown in **Supplementary Figure S10**. According to the funnel plots, the literature on the relationship between ELA and depression in childhood or adolescence was more evenly distributed on both sides of the total effect size, which suggested that there may be no publication bias in the studies. Second, the classic fail-safe N and Egger’s regression methods were examined overall (see **Table 2**). The classic fail-safe N values were sufficiently large to indicate the absence of serious publication bias (>5k+10), and the results of Egger’s regression test showed that the intercept was not significantly different from zero, which means that there was no serious publication bias in this meta-analysis.

## 4 Discussion

In terms of overall ELA, individuals exposed to ELA during childhood or adolescence were twice more likely to be at risk of depression than those not exposed to ELA, which was not entirely consistent with other studies. For example, LeMoult (7) found that ELA-exposed individuals were 2.5 times more likely to be at risk of depression than those not exposed to ELA. Similarly, Nelson et al. (111) found that any type of abuse was related to depression in adults (*OR* = 2.66). Notably, differences in outcome indicators may be due to differences in the specific forms of early adversity explored and factors such as the study population and methods, which results in differences in the fitted indicators. Considering ELA more generally seems to be a comparable environmental risk factor for depression onset in both youth and adults. While meta-analytic studies were unable to fit the cumulative effects of ELA, there was general agreement that ELA exhibited a dose-response relationship for depression in children and adolescents (112, 113).

We also examined the effects of nine different types of ELA. Emotional abuse, physical abuse, sexual abuse, emotional neglect, physical neglect, family conflict/violence, divorce, low socioeconomic status, and being left behind were associated with a significantly greater risk for depression than for youth-onset depression. The results suggested that emotional abuse was more strongly associated with depression than other forms of adversity were, which was consistent with previous research (7, 17). Emotional abuse was a more effective predictor of periodic major depression than sexual abuse, physical abuse, and neglect (114). Emotional abuse had a very prominent impact on depression in both youth and adult populations. Because emotional abuse can be a direct attack on a person’s self-worth, it is more likely to lead to negative perceptions and hopelessness depression. The non-physical forms of rejection or hostile treatment can be strongly associated with depressive disorders at different ages (115). The present study also showed that neglect (both emotional and physical neglect) can have a significant impact on depression in children and adolescents, especially emotional neglect, which is second only to emotional abuse in terms of its impact on depression (15). In terms of causes, emotional neglect and emotional abuse share the same psycho-cognitive mechanisms that may lead to feelings of powerlessness and lower self-esteem in children and adolescents, resulting in the development of emotional disorders such as depression (116).

Notably, physical abuse was more strongly associated with depression than was sexual abuse, which is consistent with the results of LeMoult (7) but different from the results of an adult population study (117). The reason for this may be that negative consequences from sexual abuse may manifest in other ways in children and adolescents, such as post-traumatic stress disorder (PTSD) (118), suicide (119), and the risk of sexual promiscuity (120). Furthermore, while the majority of offenders of physical abuse are inside the home, the majority of instances of sexual abuse occur outside of the home. Thus, physical abuse may undermine the sense of safety in the family environment more directly than sexual abuse (7), and a lack of safety can have lasting and profound effects on mood disorders in youth (121, 122).

The importance of a sense of security in the family for the development of mental health was further corroborated by domestic violence and divorce. First, the results of this study suggested that the strength of the association between domestic violence and depression was second only to that between emotional abuse and neglect. Domestic violence can directly contribute to family disharmony and is the main form of adversity that causes a high level of insecurity in children and adolescents. In addition, it is an independent and effective predictor of developmental psychopathology (123) and often coexists with other forms of adversity (124). However, the results of this study showed that divorce was less strongly associated with depression than domestic violence was, which is consistent with the divorce stress release hypothesis (125). Divorce is a stressful life event, and parental conflict is an ongoing chronic stressor for children and adolescents. If children live in an environment with frequent conflict, hostility, and even violence between parents before divorce, then parental divorce may provide children with relief from constant chronic stress, which may even have a stress-releasing effect and even alleviate depression after parental divorce (126, 127). On the other hand, this finding also reflects the importance of the sense of security brought by a harmonious family environment atmosphere for the emotional health development of children and adolescents. The results of the present study demonstrated the small effect size of divorce associated with depression in children and adolescents and the tendency for the negative effects of divorce on youth to gradually decrease over time, which further reflects the view that divorce has a limited impact (128).

This study also revealed a strong association between low socioeconomic status and youth-onset depression, but a recent meta-analytic study (7) revealed no direct association between poverty and depression in children and adolescents. There may be two reasons for this. First, the two fit different indicators, with low socioeconomic status accounting for not only income but also parental occupation and education level, especially parental education level. Research has shown that children of highly educated parents exhibit fewer mental health problems in stressful life situations (129). Additionally, the results of this study indicate that the level of development of the national economy has different effects on the relationship between low SES and youth depression. Low SES is more closely related to adolescent depression in developing countries than in developed countries. This result suggested that low SES might not be “low” in developed countries relative to developing countries and that the level of disparity between SESs is not large. Studies have shown that low SES is more strongly associated with depression in black populations than in white populations (28). In summary, the disadvantage of socioeconomic status may be an important risk factor for depression in youth, and a reduction in socioeconomic inequalities and interventions for families with low parental education might help reduce depression in youths (104, 129).

Left-behind experience refers to the prolonged separation of children from their parents the age of 16 because their parents work outside the country. Children and adolescents who suffered from being left behind have received attention from the state and scholars. The results of the meta-analysis revealed that the sample source was mainly from the central and western regions of China (mainly Chongqing, Sichuan, Anhui, and Guizhou), and the results of this study suggest that the left-behind experience is also a risk predictor of depression, which may increase emotional neglect and weaken parent-child cohesion, thus leading to depression (92). However, the effect size of being left behind during childhood and adolescence associated with depression was relatively small and tended to diminish with age. In summary, parents should try to strengthen their contact with their children and parents avoid prolonged separation from their children in the early years, which can help reduce depression and anxiety in left-behind children (130).

The above results fully illustrate that subtypes of ELA can have different degrees of impact on depression during childhood and adolescence. However, meta-analytic studies that can clarify the variability and plasticity of early adverse experiences are of greater practical value and significance. In terms of both dimensions of ELA, children and adolescents are at greater risk of depression when exposed to a poor family upbringing or domestic violence than being deprived when experiencing poverty or parental absence. The moderated analysis by year of publication revealed that the early-life adversity and threat dimensions increase in relation to depression over time, indicating that increasing attention to the impact of early-life adversity on depression in youth may also reflect that the impact of the COVID-19 pandemic could increasing the type, intensity, and duration of early-life adversity, which in turn may produce a range of mental health problems such as depression and anxiety (131, 132). However, from an intervention perspective, threat adversity is more malleable or intervening, whereas deprivation experiences such as divorce, retention, and poverty are less malleable or intervening, suggesting that positive interventions for threats may have a more important impact on improving psychopathology in youth, which has important implications for future interventions.

### 4.1 Strengths and limitations

There are several limitations in our meta-analysis. First, the sample of different potential moderating variables in this study was small and unevenly distributed, which may affect the results of the moderation analysis to some extent. Second, the present study only considered ELA in the context of unfavorable family environments originating from the family and ignored the influence of micro-systems outside the family on adolescents’ depressive relationships, such as peer isolation and bullying. Third, the majority of the studies included in this meta-analysis were cross-sectional studies, and causal inferences on the relationship between early adversity and depression could not be made for most cross-sectional studies. Fourth, according to the cumulative risk model, separate risk factors do not act individually; rather, they tend to manifest themselves in the form of clusters, but the current data do not allow us to address the cumulative effects of ELA.

Future research can be conducted in the following areas. First, the present meta-analytic study identified ELA as a risk factor for depression in children and adolescents, and we need to further explore the relationships between ELA and other affective disorders or behavioral problems. Second, we need to further examine the neuro-biological mechanisms by which exposure to ELA may increase an individual’s risk of depression and the protective mechanisms of psychological resilience resources by which exposure to ELA may decrease the risk of depression. In conclusion, focusing on the risk and protective factors for mood disorders in youths and their mechanisms of action can help children and adolescents develop emotional health. Moreover, the quality of the literature included can be improved in the future by expanding the number of databases searched and the way they are searched, facilitating in-depth analysis.

## 5 Conclusion

The present results highlight the complexity of the relationship between ELA and depression in children and adolescents. Specifically, emotional abuse was more strongly related to depression in children and adolescents than other forms of ELA. In both dimensions, threat was more closely related to depression than deprivation.

The results of this study have implications for interventions. First, educators and parents should pay close attention to threat-related forms of adversity, especially emotional abuse, which has the greatest impact on emotional problems such as depression. Conscious efforts to reduce or eliminate childhood abuse and neglect, and domestic violence and a favorable emotional climate in the familial environment are important for positive adolescent development. In addition, deprivation adversities such as low socioeconomic status, being left behind, and divorce have relatively small effects on depression in adolescents relative to threats. According to the moderating effect analysis, the effect of left-behind experience on adolescents’ depression diminishes with age, which also suggests that the older age at which children and adolescents experience deprivation, the lower their risk of depression is.

## Data Availability

The original contributions presented in the study are included in the article/**Supplementary Material**. Further inquiries can be directed to the corresponding author.
