# Supplementary material for: Depression in youths with early life adversity: a systematic review and meta-analysis
Source: Front Psychiatry. 2024 Sep 12;15:1378807. doi: 10.3389/fpsyt.2024.1378807 (PMC11424519; doi:10.3389/fpsyt.2024.1378807)
Supplement: Supplementary file 1 [file DataSheet1.zip › Search strategy.DOCX]

**Extended Data 1 – Search Strategy**

**Key Words**

- Mood Disorder OR
- Affective Disorder OR
- Depressive symptoms OR
- Depress OR
- MDD OR

*AND:*

- Child OR
- Childhood OR
- Children OR
- Adolescent OR
- Adolescence OR

*AND:*

- Adverse childhood experiences OR
- Early life adversity OR
- Early life stress OR
- Maltreatment OR
- Physical abuse OR
- Sexual abuse OR
- Emotional abuse OR
- Psychological abuse OR
- Trauma OR
- Neglect OR
- Domestic violence OR
- Divorce OR
- Socioeconomic status OR
- Left-behind OR

**Criteria**

- Limits set to English and Chinese articles (Considering that the experience of staying behind is mainly taking place in China)
- Sample must are Children and Adolescents, with depression
- Must include one or more of the nine forms of ELA(emotional abuse, physical abuse, sexual abuse, emotional neglect, physical neglect, family violence, divorce, low socioeconomic status and left-behind experience)
- Must assess the relationship between early life adversity and depression and effect sizes were directly obtained (OR, r, M±SD, etc.)
- Must be an empirical article or thesis, not including a review or meta-analysis

**Databases**

1. Web of Science 11,657 total
2. Pubmed 4974 total
3. CNKI 1868 total

Total Import: English articles 74 **selected**

Chinese articles 13 **selected**

87 total

**Measuring and defining sub-types of early life adversity**

1.**Divorce**:Children and adolescents live with one of their parents, and divorce refers to the breakup of a parent due to a bad relationship and does not include remarriage or widowhood family .

2.**Left-behind**：Children and adolescents separated from one or both parents for at least 6 months . However, if the effects of different types of staying behind on depression are explored separately in the same study, only those whose parents both have left them to work in the city, which are likely to have more severe effects, are selected.

3. **Low social economic status:**Children and adolescents from poor or low-income families.The low social status in this study is measured by objective indicators, mainly consisting of one or several indicators fitted to income, level of parental education and parental occupation.

4. **Neglect**: **Emotional neglect** issues include emotional feelings of not being able to feel cared for, loved, or feel comfortable. **Physical neglect** includes the inability to secure the provision of food, clothing, medical care, etc.

5.**Domestic Violence:** Refers to Children and adolescents living in an inhospitable family atmosphere, with frequent and normal quarrels and even violent behavior between parents, but excluding physical and psychological abuse of children by their parents.

6. **Emotional abuse:**Emotional abuse involves a pattern of failure over time on the part of a parent or caregiver to provide a developmentally appropriate and supportive environment. Abuse of this type includes the following: the restriction of movement; patterns of belittling, blaming,threatening, frightening, discriminating against, or ridiculing; and other non-physical forms of rejection or hostile treatment.

7. **Physical abuse** of a child is defined as the intentional use of physical force against a child that results in harm for the child’s health, survival, development, or dignity.

8.**Sexual abuse** is defined as the involvement of a child in sexual activity that he or she does not fully comprehend, is unable to give informed consent to, or for which the child is not developmentally prepared, or else that violates the laws or social taboos of society.

9. **Threat：**Experiences of threat involve the presence of an atypical (i.e.,unexpected) experience characterized by actual or threatened death, injury,sexual violation, or other harm to one’s physical integrity. Psychological abuse is also a form of threat in this study, and its effect on individual depression levels is even greater than that of physical abuse.

1. **Deprivation:** Experiences of deprivation involve the absence of expected environmental

inputs in cognitive (e.g., language) and social domains as well as the absence of species- and age-typical complexity in environmental stimulation.
