# Supplementary material for: Depression in youths with early life adversity: a systematic review and meta-analysis
Source: Front Psychiatry. 2024 Sep 12;15:1378807. doi: 10.3389/fpsyt.2024.1378807 (PMC11424519; doi:10.3389/fpsyt.2024.1378807)
Supplement: Supplementary file 1 [file DataSheet1.zip › Supplementary Figures.docx]

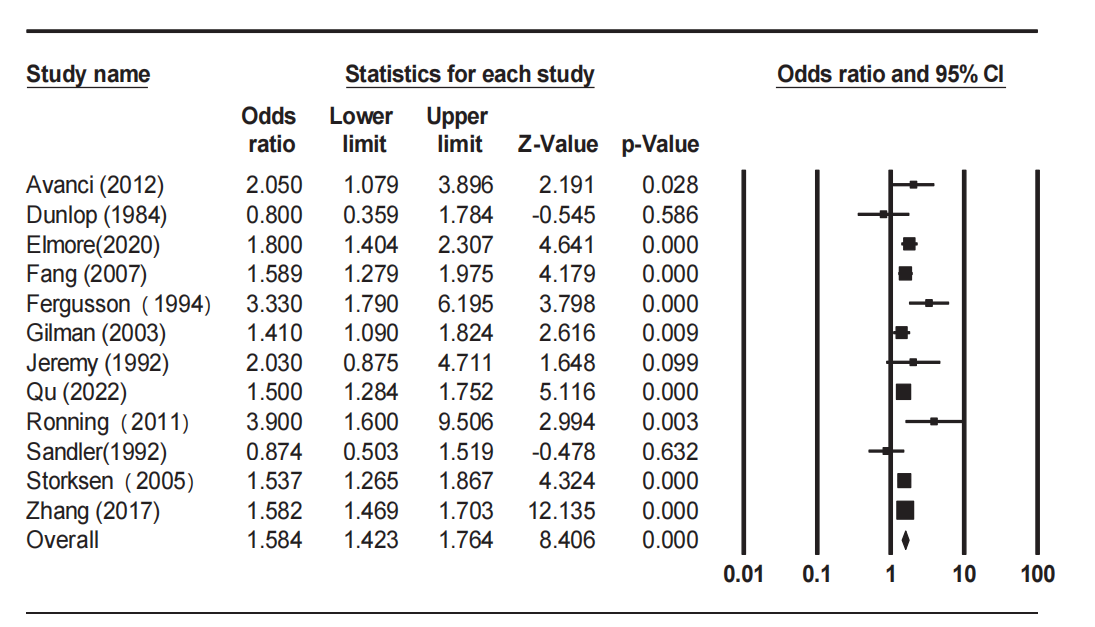


**Figure S1.** Estimated Odds Ratio for the Association Between Divorce and Depression in Childhood or Adolescence


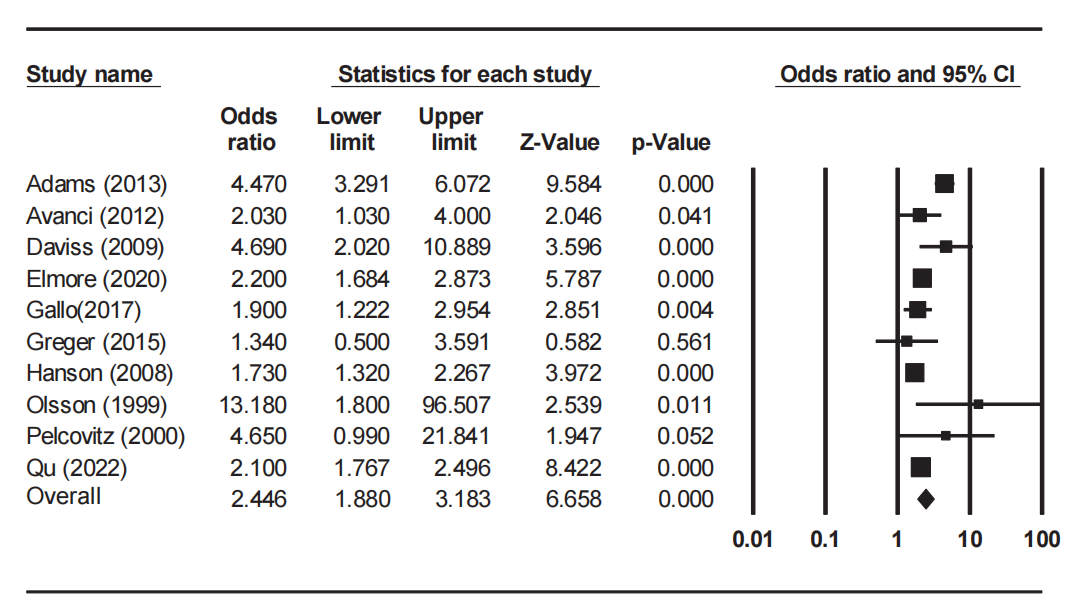


**Figure S2.** Estimated Odds Ratio for the Association Between Domestic Violence and Depression in Childhood or Adolescence


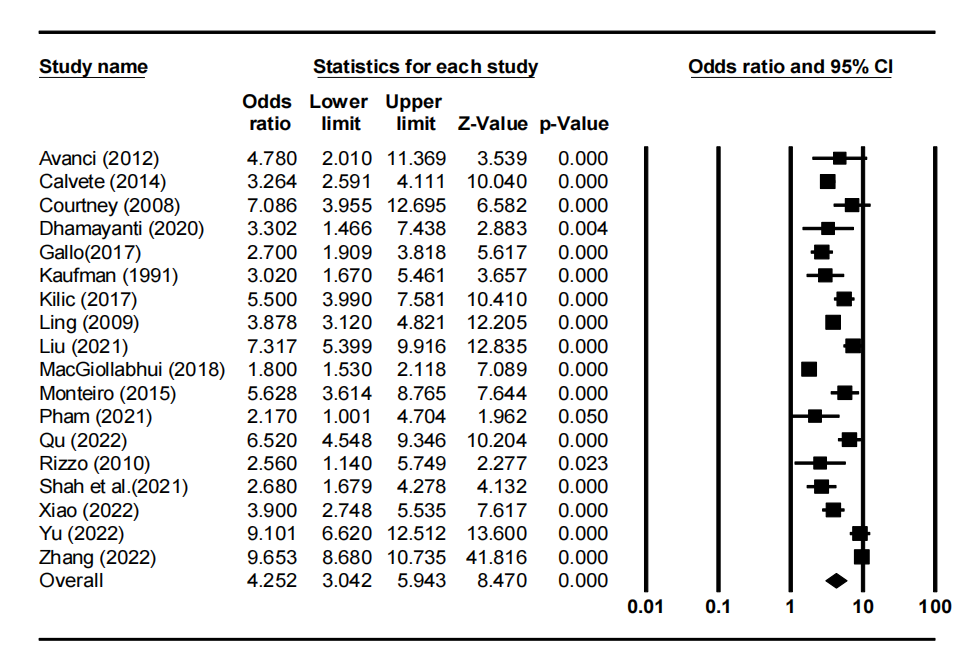


**Figure S3.** Estimated Odds Ratio for the Association Between Emotional Abuse and Depression in Childhood or Adolescence


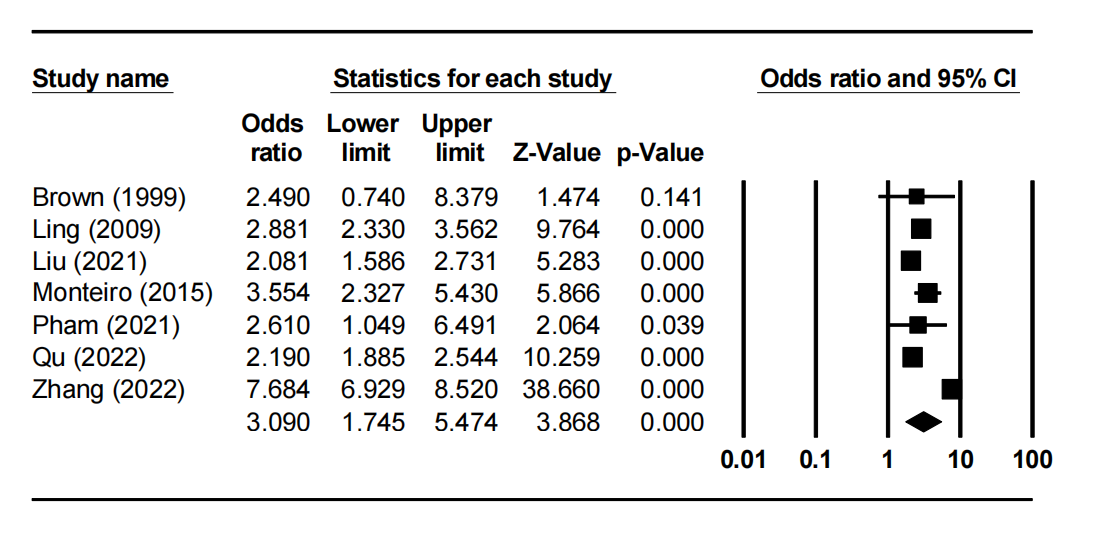


**Figure S4.** Estimated Odds Ratio for the Association Between Emotional Neglect and Depression in Childhood or Adolescence


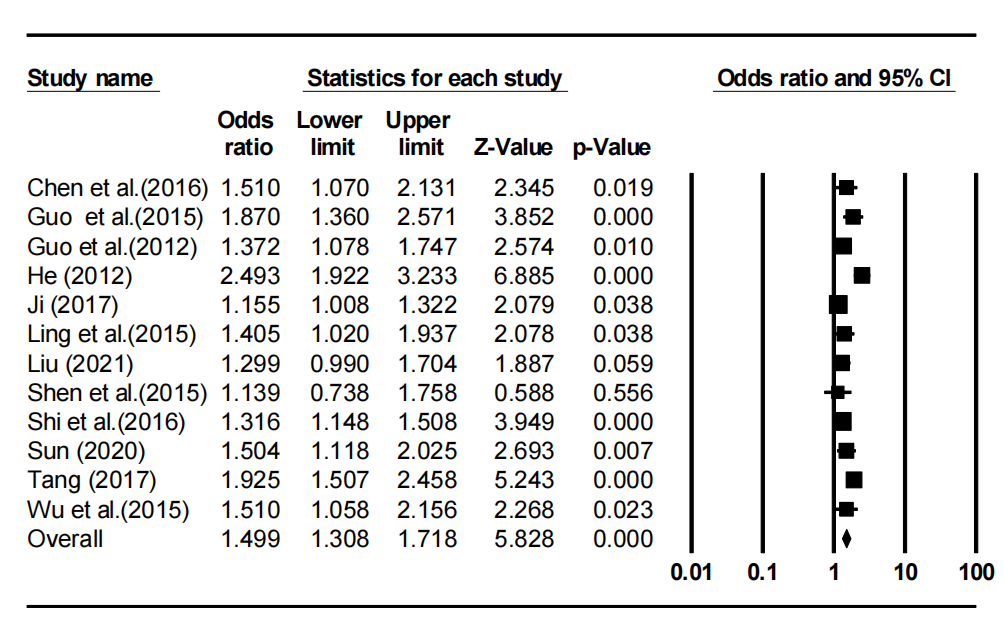


**Figure S5.** Estimated Odds Ratio for the Association Between Left-behind and Depression in Childhood or Adolescence


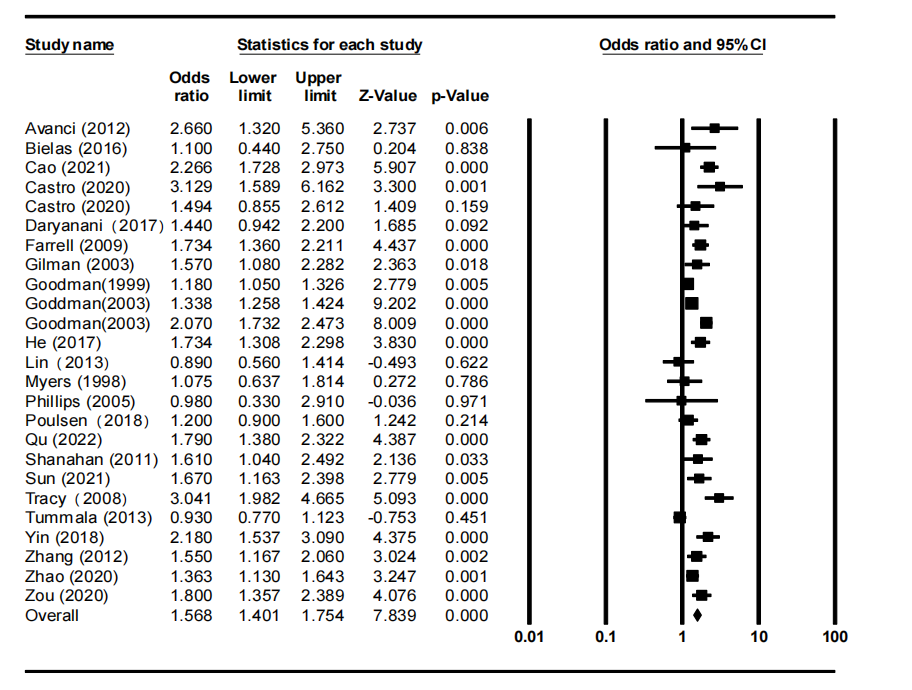


**Figure S6.** Estimated Odds Ratio for the Association Between Low So-Economic status and Depression in Childhood or Adolescence


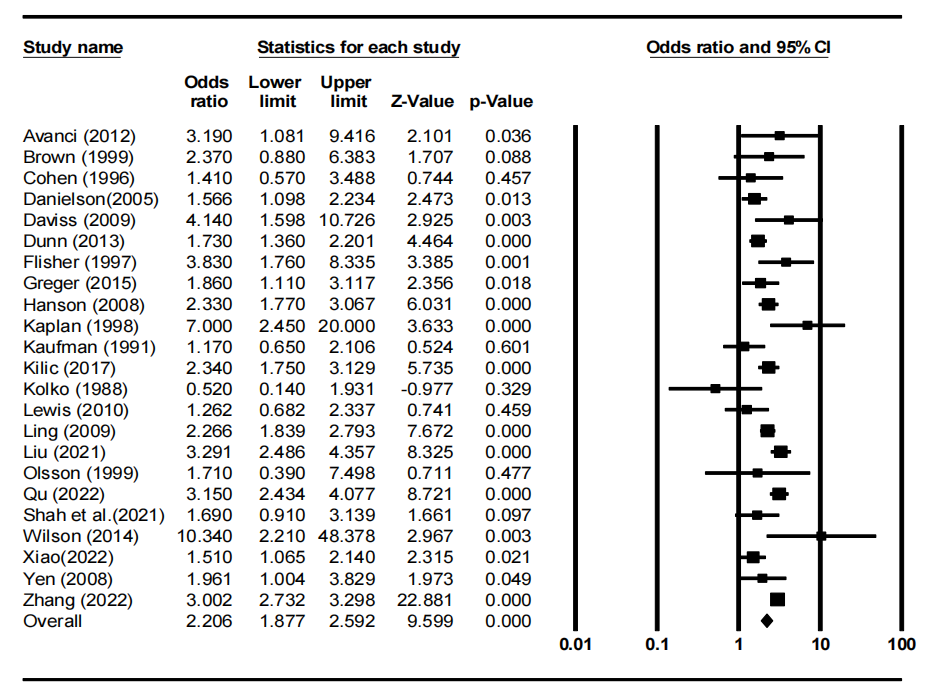


**Figure S7.** Estimated Odds Ratio for the Association Between Physical Abuse and Depression in Childhood or Adolescence


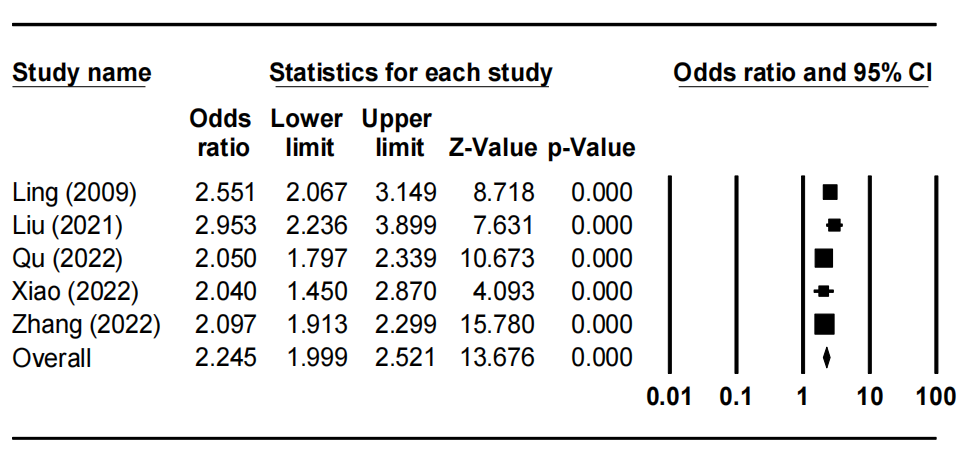


**Figure S8.** Estimated Odds Ratio for the Association Between Physical Neglect and Depression in Childhood or Adolescence


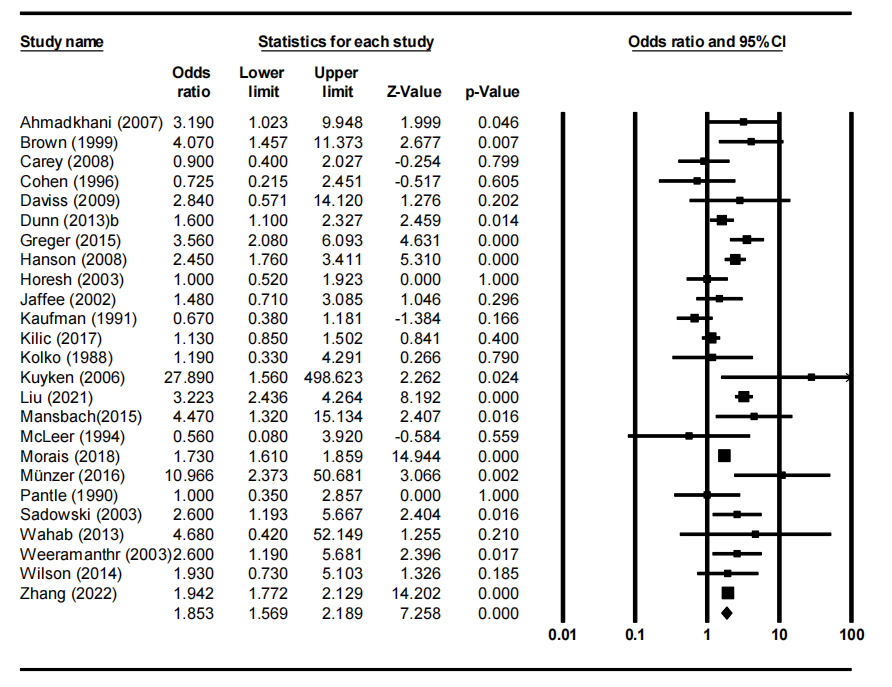


**Figure S9.** Estimated Odds Ratio for the Association Between Sexual Abuse and Depression in Childhood or Adolescence


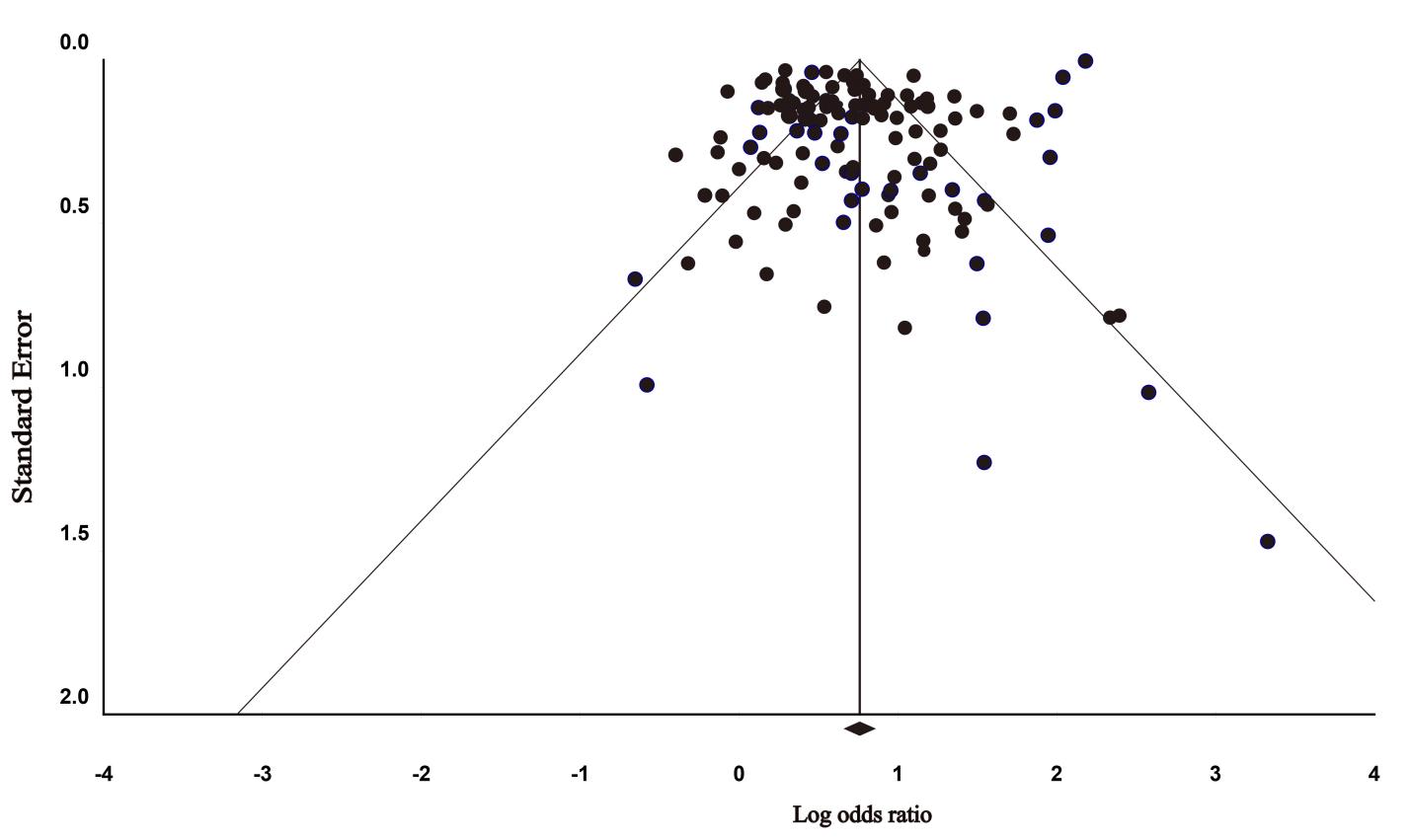


**Figure S10.** Funnel plot of research on the relationship between ELA and depression in children and adolescents
